# Arsenic exposure impels CD4 commitment in thymus and suppress T cell cytokine secretion by increasing regulatory T cells

**DOI:** 10.1038/s41598-017-07271-z

**Published:** 2017-08-02

**Authors:** Ruchi Gera, Vikas Singh, Sumonto Mitra, Anuj Kumar Sharma, Alok Singh, Arunava Dasgupta, Dhirendra Singh, Mahadeo Kumar, Pankaj Jagdale, Satyakam Patnaik, Debabrata Ghosh

**Affiliations:** 1 Immunotoxicology Laboratory, Food, Drug & Chemical Toxicology Group, CSIR-Indian Institute of Toxicology Research (CSIR-IITR), Vishvigyan Bhavan, 31 Mahatma Gandhi Marg, Lucknow, 226001 Uttar Pradesh India; 2 Academy of Scientific and Innovative Research (AcSIR), CSIR-IITR campus, Lucknow, 226001 India; 3 Microbiology, CSIR- Central Drug Research Institute, Lucknow, Uttar Pradesh India; 4 Regulatory toxicology, CSIR-Indian Institute of Toxicology Research, Lucknow, Uttar Pradesh India; 5 Water Analysis Laboratory, Nanotherapeutics and Nanomaterial Toxicology Group, CSIR-Indian Institute of Toxicology Research, Lucknow, Uttar Pradesh India

## Abstract

Arsenic is globally infamous for inducing immunosuppression associated with prevalence of opportunistic infection in exposed population, although the mechanism remains elusive. In this study, we investigate the effect of arsenic exposure on thymocyte lineage commitment and the involvement of regulatory T cells (T_reg_) in arsenic-induced immunosuppression. Male Balb/c mice were exposed to 0.038, 0.38 and 3.8 ppm sodium arsenite for 7, 15 and 30 days through oral gavage. Arsenic exposure promoted CD4 lineage commitment in a dose dependent manner supported by the expression of ThPOK in thymus. Arsenic also increased splenic CD4^+^ T cells and promoted their differentiation into T_reg_ cells. In parallel,﻿ arsenic exposure induced immunosuppression characterized by low cytokine secretion from splenocytes and increased susceptibility to *Mycobacterium fortuitum* (*M. fortuitum*) infection. Therefore, we linked arsenic-induced rise in T_reg_ cells with suppressed T_h_1 and T_h_2 related cytokines, which has been reversed by inhibition of T_reg_ cells *in-vivo* using wortmannin. Other parameters like body weight, kidney and liver function, histoanatomy of thymus and spleen as well as thymocyte and splenocytes viability were unaltered by arsenic exposure. Taken together our findings indicated that environmentally relevant dose of arsenic enhanced differentiation of T_reg_ cells which in turn induce immunosuppression in experimental animals.

## Introduction

Arsenic is a well known environmental toxicant which became globally infamous for its toxic effect in every sphere of life. The major natural source of arsenic is ground water which disseminate in the living system by different anthropogenic activity^1^. More than 200 million people globally are exposed to arsenic contaminated ground water at a level more than the permissible limit (<10 ppb)^2^. Protection against low dose of arsenic exposure is challenging because of its omnipresence therefore, the concern of health effect is shifting towards even lower doses^3^. In human and various animal models, arsenic has been found to alter immune response either by severe suppression or activation which may eventually lead to increased susceptibility to pathogen or hypersensitivity disorder respectively^4–9^. Arsenic-induced immunosuppression leads to increased susceptibility to opportunistic infections like tuberculosis as observed in arsenic exposed areas in Chile^10^. Due to infectious diseases, mortality rates were found to be increased in arsenic exposed populations^11^.

Arsenic has been reported to impair thymic development in infants concurrent with enhanced morbidity which seems to be the outcome of possible immunosuppression^12^. Arsenic exposure altered the relative distribution of different T cell subpopulation (CD4^+^, CD8^+^, T_h_1, T_h_2, T_h_17, T_reg_) in exposed population as well as *in-vitro* arsenic exposed human PBMCs^13–18^. Mitogen induced T-cell proliferative response and cytokine secretion was also found to be suppressed in arsenic exposed population^15, 19^ and *in-vitro* arsenic exposed human PBMCs^14, 16, 17^. Similar response was observed in mouse model^20–22^.

Arsenic has been shown to suppress T cell cytokine secretion by interrupting T cell receptor signaling cascade^21, 23^. However, the role of T_reg_ cells in the inhibition of T cell cytokine cannot be ignored^24, 25^. Interestingly, there have been no reports yet showing the involvement of arsenic-induced increased T_reg_ cells in the suppression of T cell cytokine secretion. Therefore, we envisage looking in to the role of T_reg_ cells in arsenic-induced suppression of T cell cytokine production.

In the present study, we examined the hypothesis that exposure to environmentally relevant dose of arsenic may increase T_reg_ cell population which in turn alter functional status of T cells leading to immunosuppression. To test this hypothesis, we investigated the effect of environmentally relevant doses of arsenic on thymocyte differentiation into CD4^+^ and CD8^+^ lineage. Subsequently, relative distribution of CD4^+^ and CD8^+^ population in spleen was checked. Functional alteration was detected by measuring T_h_1 and T_h_2 related cytokine in splenic culture supernatant. Arsenic - induced immunosuppression was validated by pathogen challenge assay using *Mycobacterium fortuitum*. We have also explored the effect of arsenic exposure on T_reg_ cells and its role in altering the T cell function. Finally, the involvement of T_reg_ cells in arsenic-induced immunosuppression was confirmed by suppressing T_reg_ cells using wortmannin *in-vivo*. To rule out the involvement of other factors; body weight, viability of thymocytes and splenocytes, blood level of AST, ALT and Urea as well as histoanatomy of thymus and spleen were checked. Here, we tried to delineate the role of T_reg_ cells in arsenic-induced suppression of T cell cytokine secretion which leads to severe immunosuppression.

## Results

### Arsenic exposure enhanced CD4^+^ T-cell lineage in thymus

**Figure 1:**
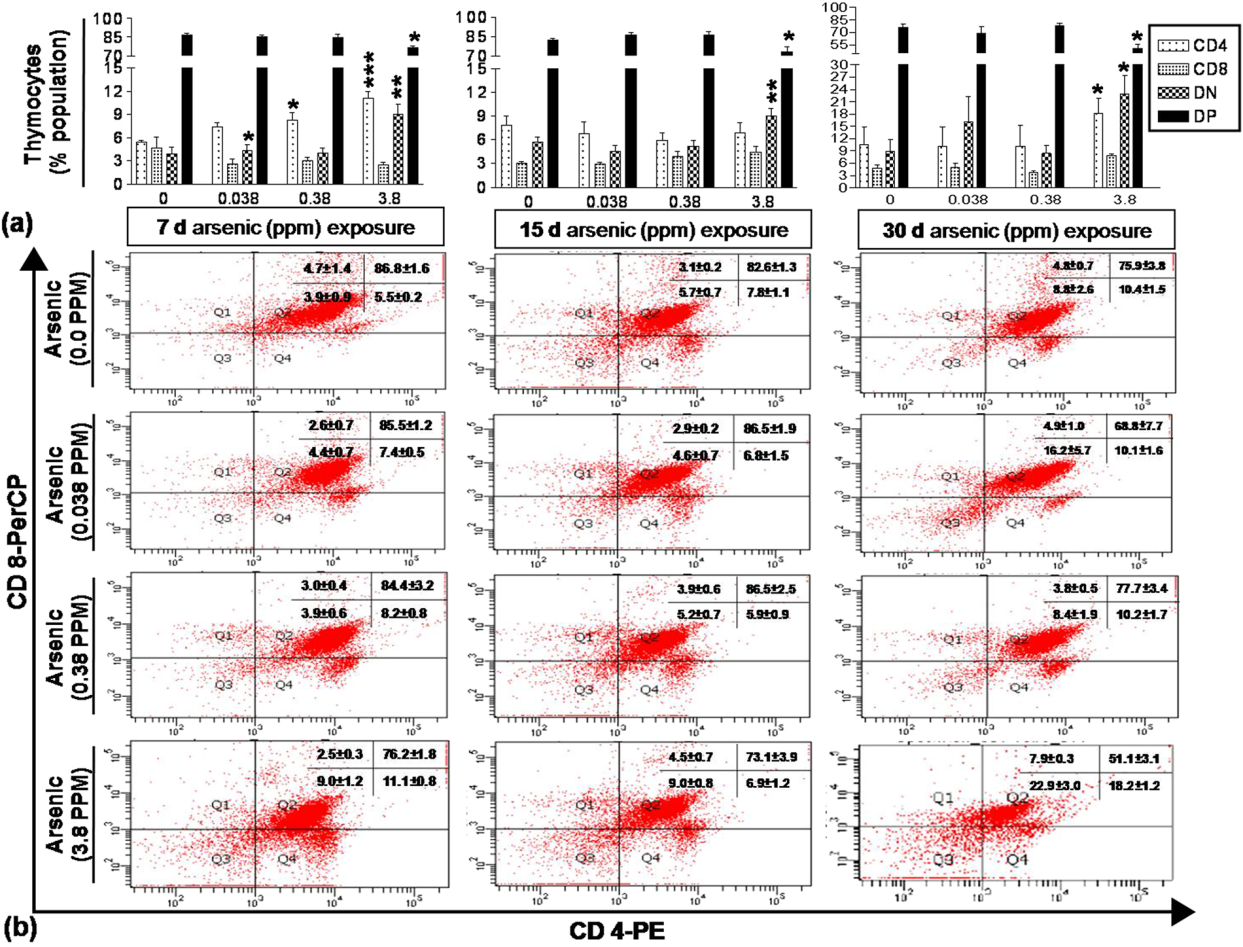
Effect of arsenic exposure on CD4^+^ T cell lineage in thymus. Thymocytes (0.2 × 10^6^) isolated from mice treated with arsenic (0.00, 0.038, 0.38 and 3.8 ppm) orally for 7, 15 and 30 days were stained with PE conjugated anti-CD4 monoclonal antibody and PerCP conjugated anti-CD8 monoclonal antibody. The PE and PerCP fluorescence were measured using flow cytometer. (**a**) Percentage of CD4^+^, CD8^+^, DN (Double negative) and DP (Double Positive) thymocytes at 7, 15 and 30 days. **(b)** Each representative dot plot reflects thymocytes stained with CD4 and CD8 antibodies. Exposure to arsenic promoted commitment of double positive thymocytes into CD4^+^ T cells. Values are mean ± S.E. (n = 9 per group). *p < 0.05, **p < 0.01 and ***p < 0.001 compared to control.

The percent distribution of CD4^−^CD8^−^ (DN: Double negative), CD4^+^CD8^+^ (DP: Double positive), CD4^+^CD8^−^ (CD4^+^) and CD4^−^CD8^+^ (CD8^+^) sub-population was determined following 7, 15 and 30 days of arsenic exposure. Following 7 day exposure to 0.038 and 0.38 ppm arsenic, no significant alteration in DN and DP sub-population was observed as compared to control group, while 3.8 ppm arsenic increased the percentage of DN cells from 3.9% (control) to 9.0% (p < 0.01) and decreased DP cells from 86.8% (control) to 76.2% (p < 0.01). The percentage of CD4^+^ T cells increased in a dose dependent manner with a significant increase from 5.5% to 8.2% (p < 0.05) and 11.1% (p < 0.01) in exposure to 0.38 and 3.8 ppm arsenic respectively for 7 days. Whereas the percentage of CD8^+^ T cells was decreased insignificantly in all arsenic exposed groups as compare to control group. Any significant alteration in CD4^+^ and CD8^+^ cells was not observed following 15days of arsenic exposure, however, 3.8 ppm arsenic increased the percentage of DN cells from 5.7% (control) to 9.0% (p < 0.05) while DP cells decreased from 82.6% to 73.1% (p < 0.05). T cell sub-population distribution did not show any significant alteration following 30 days arsenic exposure. In contrary 3.8 ppm of arsenic increased DN cells from 8.8 to 22.9 (p < 0.05) and CD4^+^ cells from 10.4 to 18.2 (p < 0.05) while decreased the DP cells significantly to 51.1 from 75.9 (p < 0.05) (Fig. 1a,b). Expression of *c-kit*, as a measure of influx of DN cells into thymus from bone marrow, was significantly increased by 3.8 ppm arsenic which corresponds to significant rise in DN cells in thymus following 30 day arsenic exposure (Supplementary Fig. S1). Interestingly, the rise in CD4^+^ cells was not due to apoptosis in CD8^+^ cells following 30 days exposure to arsenic (Supplementary Fig. S2). Therefore, exposure to arsenic resulted in influx of DN cells into thymus from bone marrow and enhanced commitment of DP cells towards CD4^+^ cell type without significant changes in CD8^+^ cells.

### Arsenic exposure altered the expression of ThPOK and RunX3 in thymus

**Figure 2:**
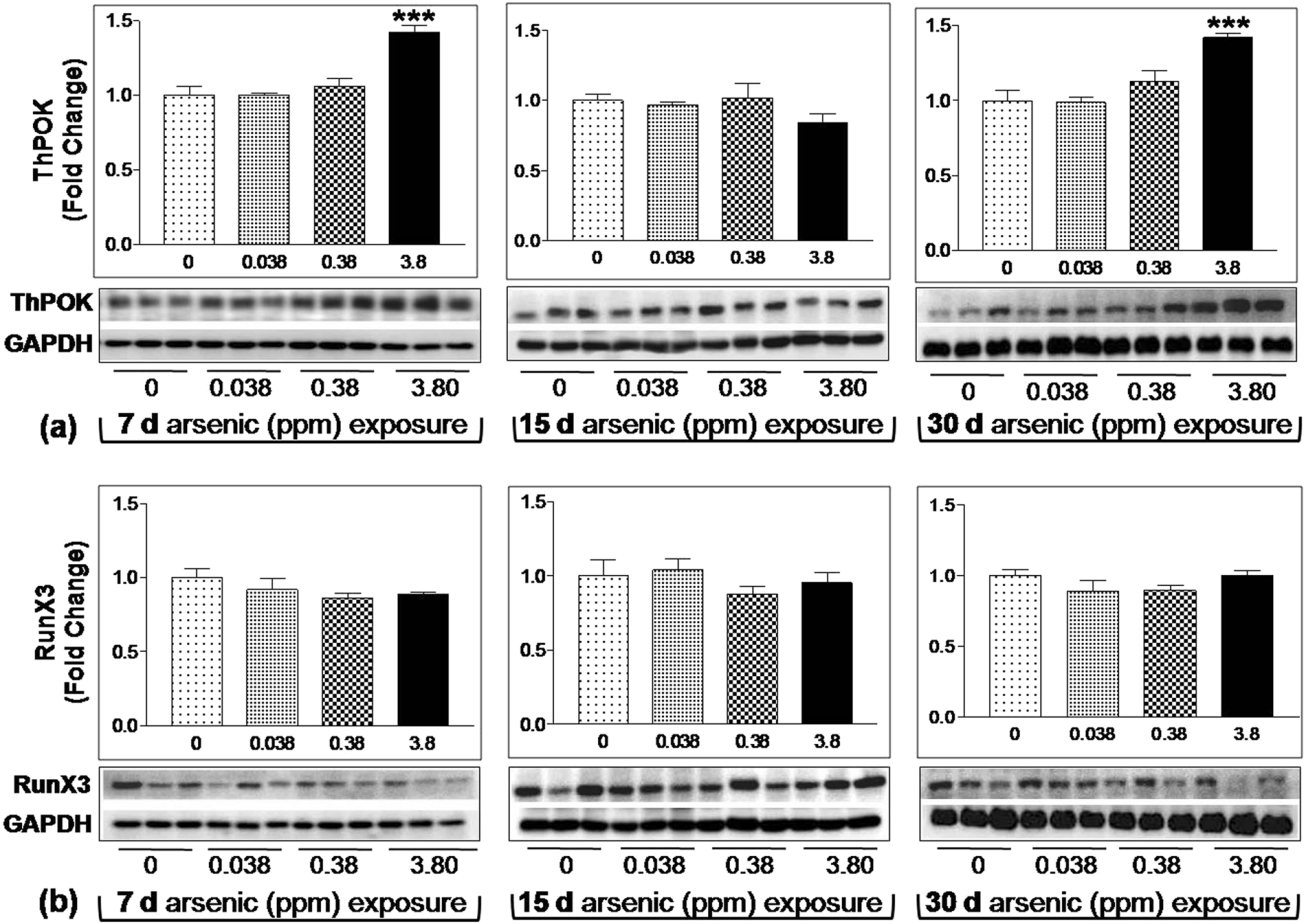
Effect of arsenic exposure on the expression of ThPOK and RunX3 in thymus. Thymus dissected from mice treated with arsenic (0.00, 0.038, 0.38 and 3.8 ppm) orally for 7, 15 and 30 days were homogenized for protein preparation and examined for expression of ThPOK (**a**) and RunX3 (**b**) transcription factors. Representative western blots and densitometric analysis (normalized by GAPDH) reflect significant rise in ThPOK expression following 7 and 30 days of 3.8 ppm arsenic exposure. Values represented as bar graph showing mean ± SE. (n = 3 per group) *p < 0.05, **p < 0.01 and ***p < 0.001 compared to control. The original full-length western blots for ThPOK, RunX3 and corresponding GAPDH are provided in supplementary information sheet from Fig. S3 to Fig. S8.

ThPOK and RunX3 are master regulator of CD4 and CD8 lineage differentiation in thymus. ThPOK promotes CD4 commitment by preventing RunX3 directed CD8 lineage differentiation in DP cells^26^. ThPOK was found to be increased significantly following 7 and 30 days of 3.8 ppm arsenic exposure (p < 0.01) while 15 days exposure group did not show any alteration (Fig. 2a). However, the expression level of CD8^+^ lineage specific transcription factor RunX3 remained unaltered throughout the exposure regimen (Fig. 2b). Alteration in expression level of both transcription factors corresponds to the immunophenotyping data shown in Fig. 1.

### Arsenic-induced enhanced CD4^+^ T cell was reflected in spleen

**Figure 3:**
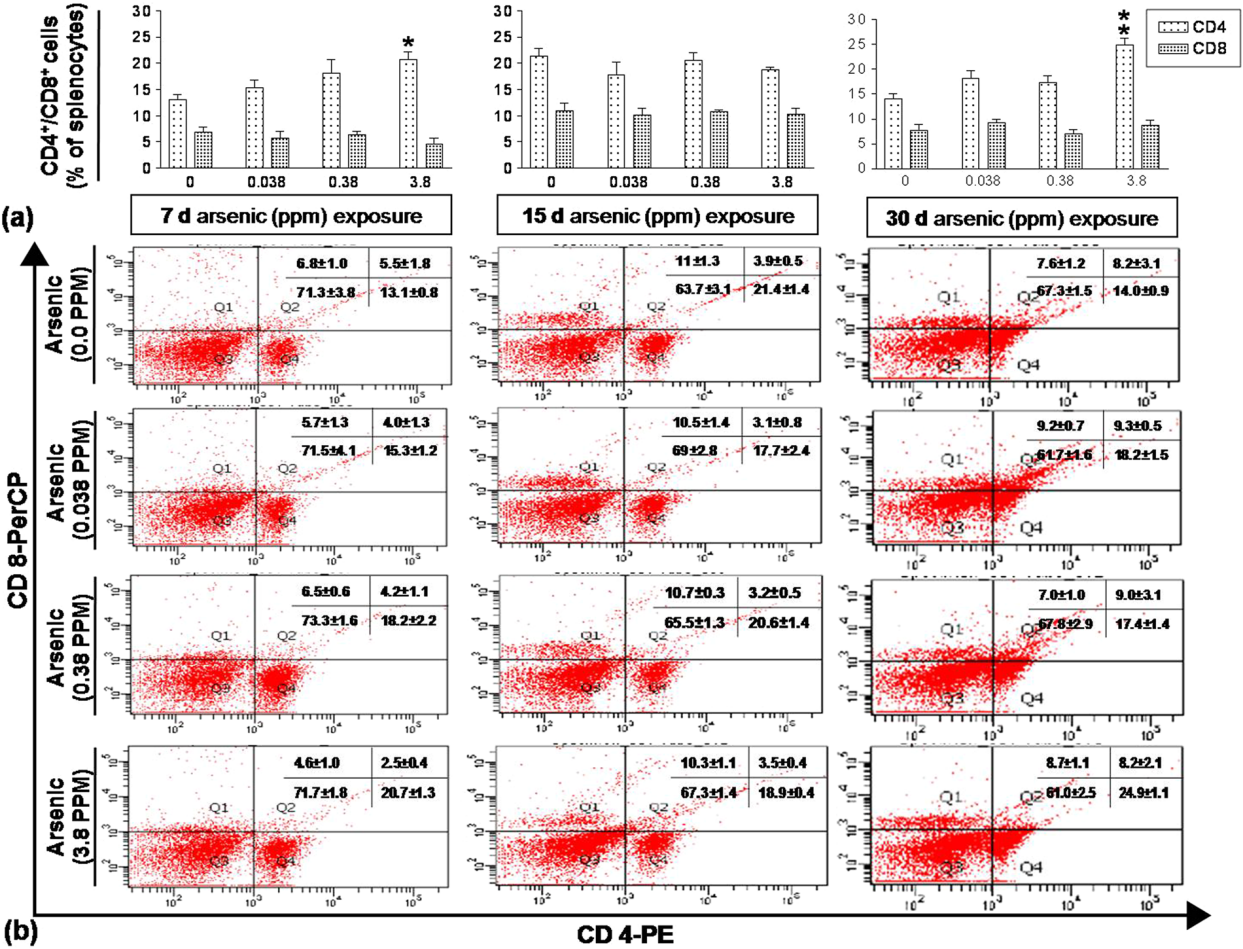
Effect of arsenic exposure on splenic CD4^+^ T cells. Splenocytes (0.2 × 10^6^) isolated from mice treated with arsenic (0.00, 0.038, 0.38 and 3.8 ppm) orally for 7, 15 and 30 days were stained with PE conjugated anti-CD4 monoclonal antibody and PerCP conjugated anti-CD8 monoclonal antibody. The PE and PerCP fluorescence were measured using flow cytometer. **(a)** Percentage of CD4^+^ and CD8^+^ T cells in splenocytes at 7, 15 and 30 days. **(b)** Each representative dot plot reflects splenocytes stained CD4 and CD8 antibodies. Arsenic promoted percent distribution of CD4^+^ T cells in spleen. Values are mean ± S.E. (n = 9 per group). *p < 0.05, **p < 0.01 and ***p < 0.001 compared to control.

At 7 day, a dose dependent increase in CD4^+^ cells was observed being significant following 3.8 ppm exposure (20.7%; p < 0.01), however, CD8^+^ population was unaffected. Following 15 days of expoures, neither of CD4^+^ nor CD8^+^ population was altered. Further exposure till 30 days increased CD4^+^ population to 24.9% in 3.8 ppm exposure group compared to 14.0% in control group (p < 0.01), whereas an insignificant increase was observed in 0.038 and 0.38 ppm exposure group. Therefore, enhanced CD4^+^ commitment in thymus had also been reflected in spleen (Fig. 3a,b).

### Arsenic exposure suppressed splenic cytokine profile

**Figure 4:**
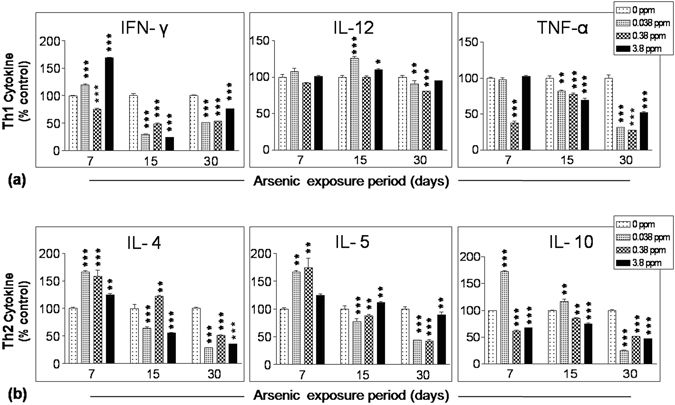
Effect of arsenic exposure on the mitogen induced splenic cytokine profile. Splenocytes (0.1 × 10^6^/200 µl/well) isolated from mice treated with arsenic (0.00, 0.038, 0.38 and 3.8 ppm) orally for 7, 15 and 30 days were incubated in 5% CO_2_ incubator at 37 °C for 72 h in the presence or absence of ConA (2.5 µg/ml). Levels of **(a)** T_h_1 (IFN- γ, IL-12 and TNF-α) and **(b)** T_h_2 (IL-4, IL-5 and IL-10) related cytokines in splenocytes culture supernatant was estimated; plated in triplicate from pooled samples of each treatment group. Fluorescence associated with different antibody-coated magnetic beads was read under Bio-Plex MAGPIX multiplex reader. The cytokine levels are expressed as percent of control. Values are mean ± S.E. (n = 3 per group). *p < 0.05, **p < 0.01 and ***p < 0.001 compared to control.

T_h_1 (IFN-γ, IL-12 and TNF-α) and T_h_2 (IL-4, IL-5 and IL-10) related cytokines were measured in ConA stimulated splenocyte culture supernatant. Arsenic exposure at all the doses and time points altered cytokine secretion to some extent. Level of IFN-γ was a little irregular following 7 days exposure but 15 and 30 days of arsenic exposure suppressed IFN-γ level in all doses significantly (p < 0.001 or <0.01). Almost same pattern was observed in T_h_1 cytokine TNF-α as well as T_h_2 cytokines IL-4 and IL-5. None of the doses affected another T_h_1 cytokine, IL-12 production significantly except 30 days exposure. T_h_2 cytokine, IL-10 was also suppressed in all the exposure groups and time points except 0.038 ppm dose in 7 and 15 days exposure groups (Fig. 4a,b). Therefore, subchronic exposure to arsenic suppressed T_h_1 and T_h_2 related cytokine production from ConA activated splenocytes.

### Effect of arsenic exposure on splenic T_reg_ cell

**Figure 5:**
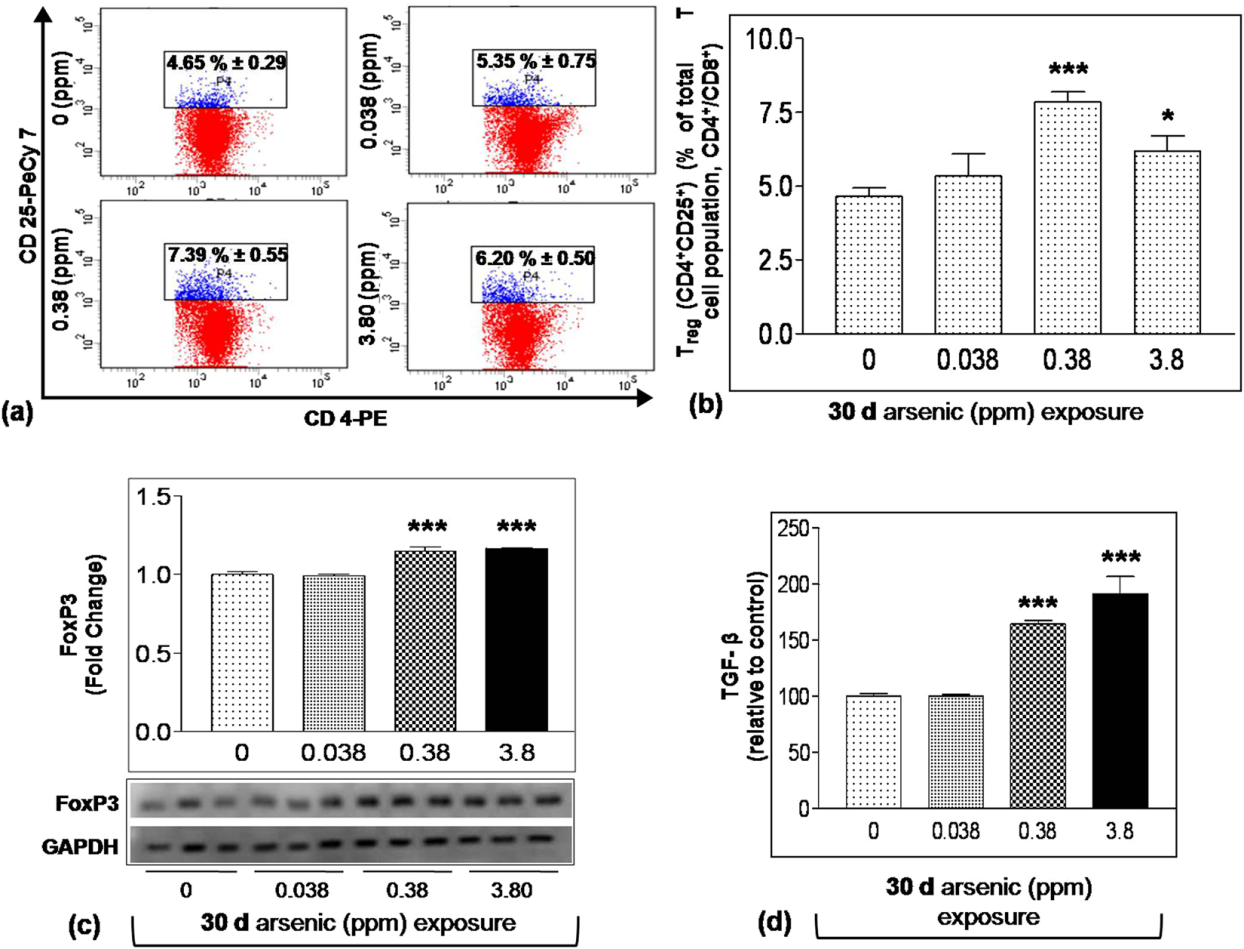
Effect of arsenic exposure on T_reg_ cell. Splenocytes isolated from mice treated with arsenic (0.00, 0.038, 0.38 and 3.8 ppm) orally for 30 days were processed to isolate CD4^+^ and CD8^+^ T cells using CD4 and CD8 specific magnetic particles by positive selection. Purity of splenic T cells was 85 to 90%. Isolated splenic T cells were stained with PE conjugated CD4, PerCP conjugated CD8 and PeCy7 conjugated CD25 antibody for 1 h at 37 °C. PE stained CD4^+^ T cells were gated for CD25^+^ cells. Each representative dot plot **(a)** and bar graph **(b)** reflects percent population of CD4^+^CD25^+^ T cells in total parent T cell population. **(c)** Representative western blot and densitometric analysis showing level of FoxP3 in spleen dissected from each treatment group. **(d)** Levels of TGF-β in splenocyte culture supernatants expressed as percent of control. Values are mean ± S.E. (n = 3-5 per group). *p < 0.05, **p < 0.01 and ***p < 0.001 compared to control. The original full-length western blots for FoxP3 and GAPDH are provided in supplementary information sheet in Fig. S9.

To investigate the possible reason for arsenic-induced T cell cytokine suppression, splenic T_reg_ cells distribution was studied in 30 days arsenic exposed groups. Interestingly, T_reg_ cell population (CD4^+^CD25^+^) was found to be increased from 4.65% to 5.35% in 0.038 ppm exposure group and significantly (p < 0.01) increased to 7.39% and 6.2% following 0.38 and 3.8 ppm of arsenic exposure (Fig. 5a,b). Increased level of T_reg_ cells in spleen was confirmed by measuring transcription factor FoxP3 expression,which controls T cell differentiation into T_reg_ cells^27^. Significant rise in FoxP3 expression was observed in spleen of 0.38 and 3.8 ppm exposed group (p < 0.001) (Fig. 5c). Level of T_reg_ cell associated cytokine TGF-β in ConA stimulated splenocyte culture supernatant was measured to be significantly higher in 0.38 and 3.8 ppm arsenic exposed groups (p < 0.001) (Fig. 5d).

### Inhibition of T_reg_ cells reversed arsenic-induced cytokine suppression

**Figure 6:**
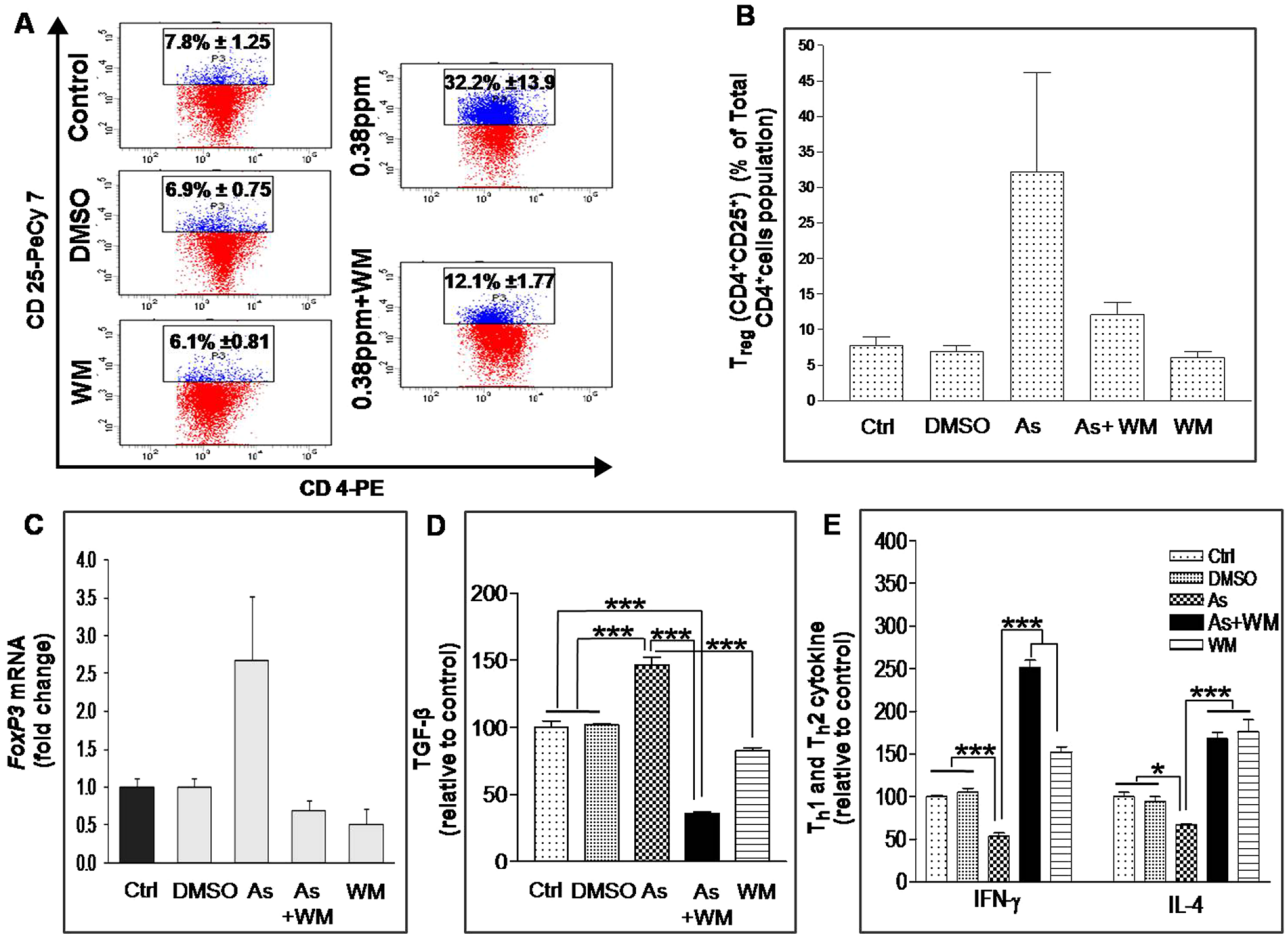
Effect of wortmannin on arsenic-induced altered splenic cytokine profile. Splenocytes isolated from mice treated with DMSO as vehicle, 0.38 ppm arsenic, 0.38 ppm arsenic along with wortmannin and only wortmannin group were processed to isolate CD4^+^ T cells using CD4 magnetic particles by positive selection. Isolated CD4^+^ T cells were stained with PE conjugated CD4 and PeCy7 conjugated CD25 antibody for 1 h at 37 °C. Each representative dot plot **(A)** and Bar graph **(B)** reflects percent population of CD4^+^CD25^+^ T cells in total parent CD4 population. **(C)** *FoxP3* transcript level in isolated CD4^+^ T cells. **(D)** Levels of TGF-β, **(E)** IFN-γ and IL-4 in splenocyte culture supernatant expressed as percent of control. Values are mean ± S.E. (n = 3–5 per group). *p < 0.05, **p < 0.01 and ***p < 0.001 compared to control. Ctrl; Control; DMSO; Dimethyl sulfoxide; As; 0.38 ppm arsenic; WM; Wortmannin.

To confirm the involvement of T_reg_ cells in T cell cytokine suppression upon arsenic exposure, we inhibited T_reg_ population by *in-vivo* wortmannin treatment. We found that arsenic-induced rise in splenic T_reg_ population from 7.8% to 32.2% was restricted to 12.1% following wortmannin treatment in 30 days arsenic exposed group (p > 0.05). However in wortmanin alone treated group, T_reg_ population decreased to 6.1% as compare to control group (p > 0.05) (Fig. 6A,B). Viability of splenocytes from different exposure groups was found to be unaltered (data not shown). T_reg_ inhibition by wortmanin was confirmed by examining significantly decreased *FoxP3* mRNA expression in arsenic exposed splenic T cells (Fig. 6C). In arsenic exposed mice, inhibition of T_reg_ cells was associated with suppressed TGF-β production from ConA activated spenocytes as compared to control (p < 0.001) and arsenic exposed group (p < 0.001) (Fig. 6D). Inhibition in T_reg_ cells and TGF-β cytokine production in arsenic exposed group resulted in significantly high secretion of IFN-γ and IL-4 cytokine compared to control and only arsenic treated group (p < 0.001) (Fig. 6E).

### Effect of arsenic exposure on pathogen clearance

**Figure 7:**
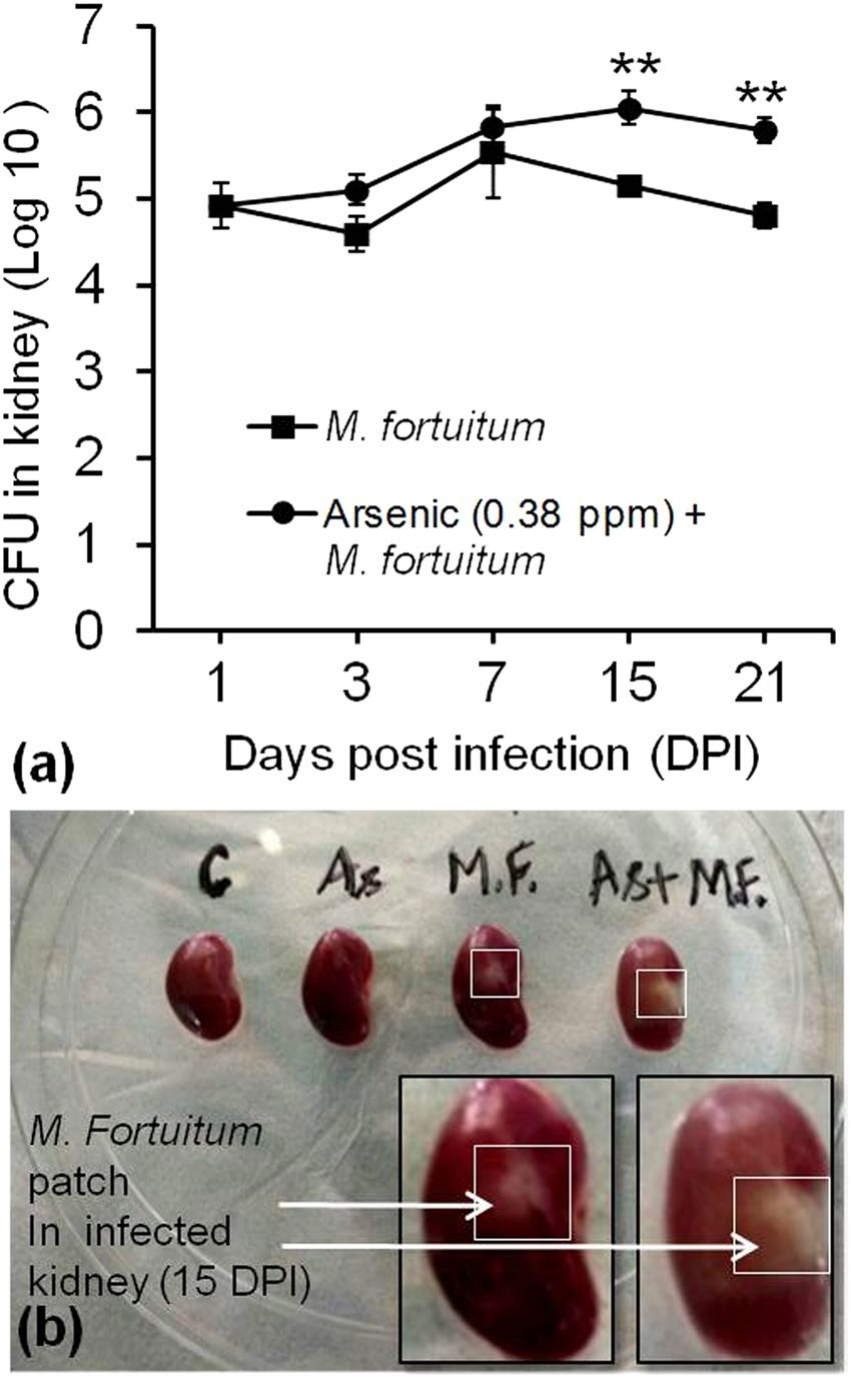
Effect of arsenic exposure on pathogen clearance. Mice exposed to 0.38 ppm arsenic for 30 days were infected with 10^7^ of pathogen and sacrificed following 3, 7, 15 and 21 days post infection (DPI). Dissected kidney from infected mice were homogenized and plated for colony forming unit (CFU) estimation. **(a)** The number of *M. fortuitum* CFU in kidney represented as mean ± S.E of Log_10_ (n = 3 per group). **(b)** Representative photomicrographs of visible *M. fortuitum* patches on kidney following 15DPI. *p < 0.05, **p < 0.01 and ***p < 0.001 compared to M. fortuitum group.

To validate arsenic - induced immune suppression, we challenged 30 day 0.38 ppm arsenic exposed mice with *M. fortuitum*. Bacterial load in kidney was persistently higher following 3, 7, 15 and 21 DPI as compared to arsenic unexposed group. In arsenic exposed group, CFU increased from 4.92 Log_10_ at 1 DPI to 6.05 Log_10_ after 15 DPI and found to be higher than arsenic unexposed group which was 5.15 Log_10_ at 15 DPI (p < 0.05). Following 21 DPI, CFU in arsenic exposed group reduced to 5.79 Log_10_ which was significantly higher than arsenic unexposed group with 4.8 Log_10_ (p < 0.01) CFU (Fig. 7a). Rise in bacterial load also reflected morphologically in the form of prominent and large white patches of *M. fortuitum* infection on the kidney of arsenic exposed group following 15 DPI as compared to light and small patches on the kidney of arsenic unexposed group (Fig. 7b).

### Effect of arsenic exposure on general parameters

**Figure 8:**
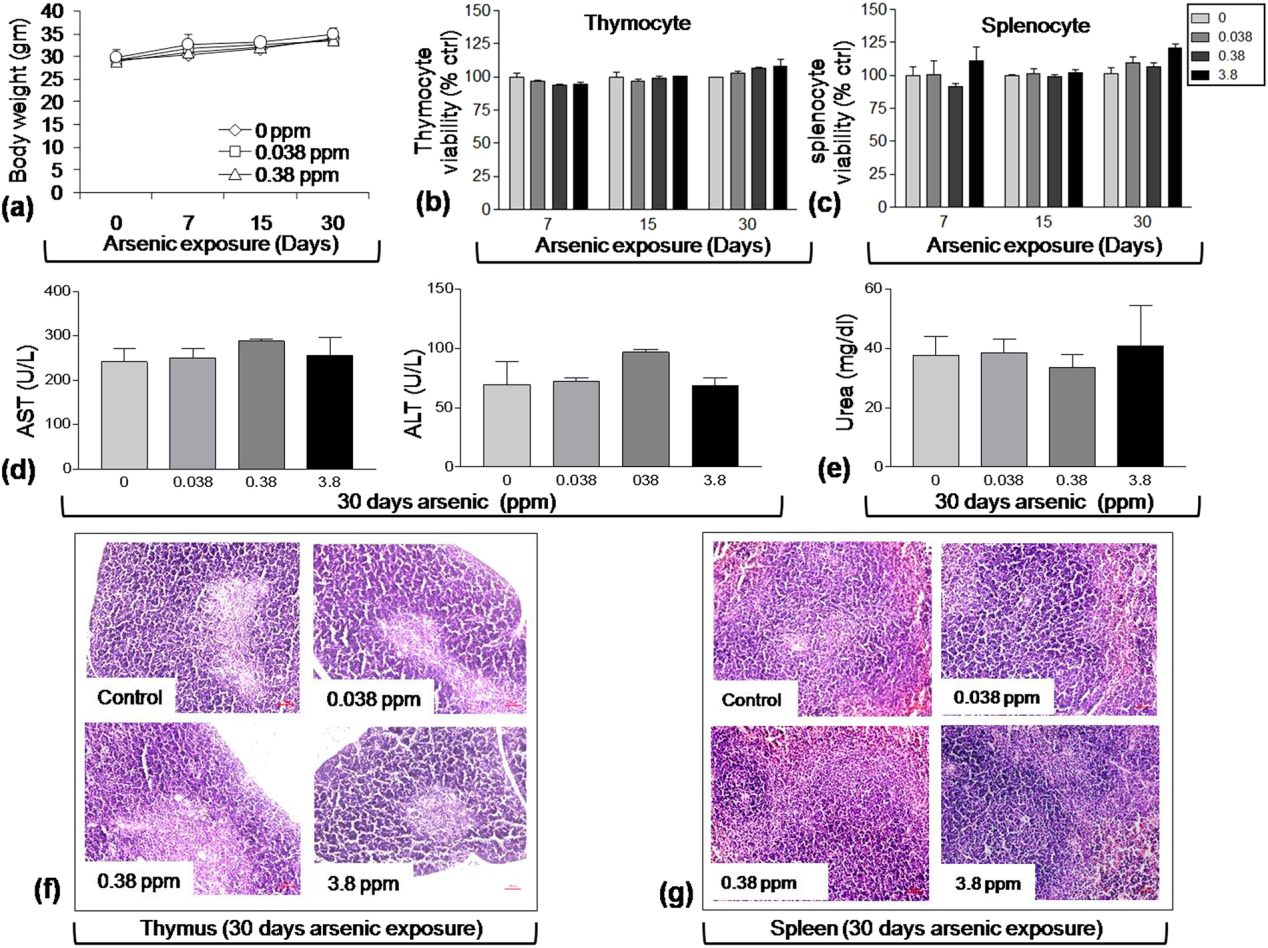
Effect of arsenic exposure on general parameters. Following arsenic exposure mice body weight was recorded weekly. **(a)** Line graph represents the changes in body weight. Values presented as mean ± S.E at each time point (n = 5–8 per group). **(b)** Thymocytes and **(c)** splenocytes (0.1 × 10^6^ cells/200 µl/well) isolated from mice treated with arsenic (0.00, 0.038, 0.38 and 3.8 ppm) orally for 7, 15 and 30 days were cultured at 37 °C in 5% CO_2_ incubator and viability was checked following 72 h incubation using Presto blue reagent. Fluorescence was measured at 544 (ex.) and 610 (em.) expressed as percent of control (n = 3–5 per group). Serum level of **(d)** AST, ALT and **(e)** urea expressed as mean ± S.E. (n = 3 per group). Representative micrographs of histo-architecture of **(f)** thymus and **(g)** spleen obtained at 10x magnification (scale bar = 60 µm) from control and 30 day exposed groups.

***Body weight*** **:** Exposure of mice to arsenic resulted in no significant changes in body weight of mice with time. In the control group receiving water alone, body weight increased from 29.3 ± 1.3 to 33.9 ± 1.7 g. All the arsenic exposed groups showed similar changes in body weight with time as in control (Fig. 8a). ***Lymphocyte viability:*** Following 72 h *ex-vivo* culture of thymocytes and splenocytes, no significant changes in viability was observed in any of the arsenic exposure group compared to control group (Fig. 8b,c). ***Liver and kidney function:*** Serum level of AST, ALT (liver function) and Urea (Kidney function) were estimated. No significant alteration was observed between control and different exposure group following 30 days (Fig. 8d,e). ***Thymic and splenic histopathology:*** Exposure to arsenic was not found to affect histo-architecture of thymus and spleen by any dose of arsenic upon 30 days of exposure. No major difference in the histoarchitecture was observed between control and exposed group (Fig. 8f,g).

## Discussion

The present study was carried out in male mouse model to avoid gender specific difference in immune response^28^. However, the use of mouse model has always been questioned for human relevance. Since mice evolved in a quite different environment to humans and have been exposed to different antigens and their immune systems might therefore be expected to have evolved in subtly different ways but the basic development and differentiation process of immune cells is similar^29^. Although they have fine differences but exhibit profound functional similarities in immune response to xenobiotics thereby making it a good model system to study immune response which can be judiciously extrapolated for human concern^20–22^.

In the present study we aimed to check whether low level arsenic exposure can alter the differentiation of thymocytes into CD4/CD8^30^. We also intended to study whether alteration in T cell differentiation has any role in arsenic - induced immunosuppression, which is yet to be explained clearly. Alteration in thymocyte differentiation may affect the balance in the ratio of different subset of T cells in the peripheral system and thereby can influence the immune response^31^. Therefore, arsenic associated immunotoxic effect is of important concern. To our knowledge, this is the first report demonstrating the effect of arsenic exposure on CD4/CD8 differentiation in thymus. Although few studies reported xenobiotic-induced alteration in thymic lymphocyte composition resulted from death of thymocytes^32, 33^, but the present study showed that arsenic altered thymocyte differentiation without affecting its viability or histoanatomy of thymus.

Arsenic exposure promoted CD4 lineage differentiation without any major alteration in CD8 population. High influx of DN cells replenished the pool of DP population in thymus following enhanced differentiation towards CD4^+^ cells. Changes observed in percent distribution of thymocyte subsets following 7 days of arsenic exposure were not visible after 15 days of exposure. Continuous arsenic exposure rules out the possible involvement of arsenic deprivation for the observed recovery in 15 days arsenic exposed group. Therefore, some active process, which we are not sure about, may be involved in bringing back the regular thymocyte distribution. However, the homeostatic regulation was disturbed again following 30 days of arsenic exposure resulting in a significantly high production of CD4^+^ cells and high influx of DN cells in thymus in 3.8 ppm exposed group (Fig. 1).

This was further confirmed by examining the expression pattern of CD4 and CD8 lineage specific transcription factors ThPOK and RunX3 respectively^34^. Higher expression of ThPOK following 3.8 ppm arsenic at 7 and 30 day of exposure supported the increased differentiation into CD4^+^ population in thymus. In contrary, the unaltered expression of RunX3 supported the unaffected CD8 population (Fig. 2). These observations highlighted that the process of thymocyte differentiation into CD4^+^ or CD8^+^ in thymus is very sensitive to arsenic.

Once DP cells differentiated into either CD4^+^ or CD8^+^ cells they reach secondary lymphoid organs for further maturation process. Therefore, percent distribution of CD4^+^ and CD8^+^ cells were analyzed in spleen following arsenic exposure. Higher CD4^+^ population was reflected in spleen following 7 and 30 days of arsenic exposure (Fig. 3). However, low CD4^+^ population in splenic lymphocytes was reported following 30 days exposure of 1 ppm arsenic to male CD57BL6N mice^21^. Therefore, the differential response of CD4^+^ cells may be due to either dose difference or species specificity^21, 35, 36^.

Arsenic has been shown to affect T_h_1 associated cytokines in arsenic exposed human lymphocytes^1, 37^. In our study, 7 days exposure to low dose of arsenic (0.038 and 0.38 ppm) promoted T_h_2 related cytokines in general which might reflect asthma and allergic diseases like symptoms^38^. In contrast, higher dose of arsenic (3.8 ppm) induced T_h_1 response which might contribute in the induction of autoimmune diseases like symptoms^39^. Following 15 days of arsenic exposure, cytokine production exhibited a decreasing pattern compared to 7 days exposed group except IL-12. Since percent distribution of CD4^+^ cells was not altered following 15 day arsenic exposure therefore, reflecting a time and dose dependent functional suppression of T cells^40^. Longer exposure to arsenic for 30 days however enhanced CD4^+^ population in spleen but interestingly altered their functional status which has been reflected in drastically reduced secretion of T_h_1 and T_h_2 related cytokines from ConA-activated splenocytes (Fig. 4). This finding is consistent with previous studies showing suppressed production of IFN-γ, IL-4, IL-5, IL-10 and TNF-α in arsenic exposed human population as well as in mice model^15, 19, 21, 41^. Reduced secretion of cytokines may result in inefficient immune response against pathogen which may enhance the possibility of opportunistic infections. Previous studies have also reported high risk for development of respiratory tract infections^8, 42^, chronic lung diseases^27^ in arsenic exposed population. Therefore, elucidating the mechanism of immunosuppression may help intervening the arsenic-induced disease susceptibility. However, defective T cell receptor signaling cascade in response to arsenic exposure may lead to suppressed cytokine production^21^ but the involvement of T_reg_ cells in the suppression of cytokine production cannot be ignored^24, 25^.

In the present study, we found that 30 day exposure to arsenic (0.38 ppm and 3.8 ppm) increased number of splenic T_reg_ cells expressed as CD4^+^CD25^+^ cells and FoxP3 expression (Fig. 5a–c). Similar increase in T_reg_ cells following arsenic exposure *in - vitro* ^43^ as well as higher expression of T_reg_ differentiation supporting IL-2RB gene in arsenic exposed lymphocytes had been observed^23, 44^. Unlike T_h_1/T_h_2 related cytokines, exposure to arsenic enhanced TGF-β secretion from activated splenocytes (Fig. 5d) which stimulates T_reg_ cell differentiation by promoting FoxP3 expression^45^ and simultaneously inhibits other cytokine production^46^.

Therefore, most likely the suppressive effect of arsenic is associated with the formation of high T_reg_ cells and consequent higher level of TGF-β secretion. Involvement of T_reg_ cells in the event of cytokine suppression was confirmed by inhibiting T_reg_ production in mice by targeting PI3K-AKT pathway using wortmannin^47^. Since 30 days exposure to 0.38 ppm arsenic increased T_reg_ population significantly hence we selected this group for T_reg_ reversal study. Interestingly, wortmannin treatment significantly brought down the arsenic-induced increased T_reg_ population, *FoxP3* mRNA expression in splenic T cells and TGF-β production from splenocytes. Inhibition of T_reg_ cells led to enhanced production of typical T_h_1 cytokine IFN-γ & T_h_2 cytokine IL-4 in arsenic exposed splenocytes culture supernatant. These results confirmed the participation of T_reg_ cells in suppressing cytokine secretion following arsenic exposure (Fig. 6). These findings made us to conclude that environmentally relevant concentration of arsenic induced immunosuppression by increasing T_reg_ cells in mice.

Arsenic-induced immunosuppression may promote infectious diseases like tuberculosis^10^. In our study, the immunosuppression was validated using *M. fortuitum*. It is a non tubercular opportunistic pathogen, but may become infectious under immune compromised state. Growth kinetics and spread of *M. fortuitum* in mouse model is well studied following tail vein injection^48^ and it is well accepted as an alternative model pathogen and injection through tail vein is a regular process in experimental scenario. Once it reaches blood it will spread and infect an immune compromised subject^49, 50^. In the present study, increased bacterial load reflected high persistence of pathogen due to inefficient clearance from kidney (Fig. 7)^51^. Longer survival of pathogen can be due to impaired macrophage function as reflected by suppressed TNF-α and IL-12 production from arsenic exposed splenocytes as shown in (Fig. 3)^52, 53^. Ineffective macrophage induction for TNF-α and IL-12 production can be resulted from low level of IFN-γ secretion from T cells following arsenic exposure^54^. These results confirmed that suppressed cytokine production from splenocytes following arsenic exposure induced immuosuppression which enhanced the prevalence of opportunistic infections^7, 55^ and corroborate the evidences of rising tuberculosis rate with high arsenic exposure levels in Chile^10^.

To rule out the possibility of involvement of other factors in the event of arsenic-induced immunosuppression alteration in the body weight, thymic and splenic viability, histoarchitecture as well as liver and kidney function were tested following arsenic exposure. None of the parameters were found to be altered significantly compared to control group, thereby confirming the noninvolvement of other physiological parameters (Fig. 8).

**Figure 9:**
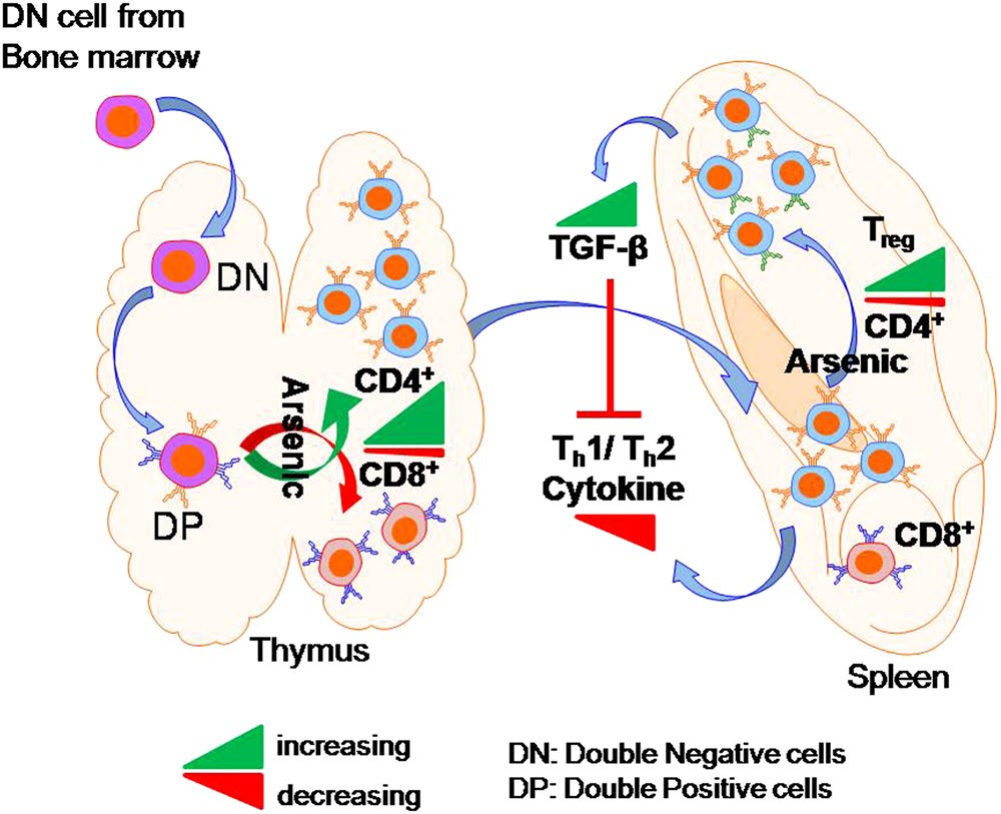
Schematic representation of arsenic-induced T_reg_ cells mediated immnosuppression. Exposure to arsenic promoted commitment of DP cells into CD4^+^ T cells in thymus. Simultaneously arsenic enhanced differentiation of splenic CD4^+^ T cells into T_reg_ cells. Increased T_reg_ cells secreted high amount of TGF-β which subsequently inhibited T_h_1 and T_h_2 related cytokines production thereby leading to immunosuppression.

In conclusion, the present study clearly showed that exposure to environmentally relevant doses of arsenic disturb thymocyte differentiation as reflected by enhanced commitment into CD4 lineage in thymus. It has also been clearly demonstrated that arsenic exposure affected T cell cytokine secretion by up regulating T_reg_ population thereby induced immunosuppression (Fig. 9). Since mycobacterium is known to exploit T_reg_ cell mediated immune suppression for their survival^56^ therefore these results also direct us to understand the participation of T_reg_ cells in supporting mycobacterium survival and increasing the risk of mycobacterail disease in arsenic exposed individuals^10^.

## Methods

### Reagents and antibodies

Cell culture and real time PCR reagents were purchased from thermo fisher scientific. Fluorochrome tagged antibodies for flowcytometry and T cell isolation kit were procured from BD Bioscience. Antibodies and reagents for cytokine multiplex assay were from millipore. Sodium arsenite (NaAsO_2_) and other general laboratory reagensts were procured from Sigma Aldrich.

### Animals and arsenic exposure

Four to five weeks old male Balb/c mice were procured from the CSIR-Indian Institute of Toxicology Research (CSIR-IITR) animal facility. All The protocol for the study was approved by the Institutional Animal Ethics Committee of CSIR-IITR, Lucknow, India, and all experiments have been carried out in accordance with the guidelines laid down by the committee for the purpose of control and supervision of experiments on animals (CPCSEA), Ministry of Environment and Forests (Government of India), New Delhi, India. Mice were housed in ventilated polypropylene cages with food and water supplied ad libitum and acclimatized for one week (25 ± 2 °C room temp with 12 h day light cycle) before arsenic exposure started. Animals were divided into 12 groups containing 15 animals (5 mice in each cage) in each group (0.0, 0.038, 0.380, 3.800 ppm sodium arsenite and three time points: 7, 15, 30 days). Sodium arsenite was dissolved in distilled water and the treatment given daily afternoon through oral gavage (≈100 μl). Among them 0.38 ppm is most frequently found in public water sources in India^57^ and being used in other studies^58^. Weights of mice were monitored weekly to examine changes in body weight. Following arsenic exposure regimen animals were euthanized using chloroform.

### Wortmanin treatment

Mice were intraperitoneally injected with 40 µg wortmanin diluted in 100 μl PBS from a stock solution (4 mg/ml in DMSO). Wortmannin given every alternate day starting from day 24 upto day 30 of arsenic exposure^47^.

### Thymic and splenic cells preparation

Thymic and splenic single cell suspension was prepared following standard method, resuspended in complete medium (RPMI 1640, 2 mM glutamine, 1% antibiotic-antimycotic solution 10% heat inactivated FBS, 50 µM β-mercaptoethanol, 25 mM HEPES, 25 mM glucose, 1 mM Sodium pyruvate) and enumerated using hemocytometer.

### Cell viability assay

Thymocytes and splenocytes were seeded at a density of 0.1 × 10^6^ cells/200 µl/ well in 96 well culture plates and incubated in a 5% CO_2_ incubator at 37 °C for 72 h. Cell viability was determined using Presto blue reagent (Life Technologies) following manufacturer’s protocol in a microplate reader (FLUOstar Omega, BMG).

### Flow cytometric analysis of T lymphocytes

Thymocytes and splenocytes (0.2 × 10^6^) were stained in 200 µl PBS with PE-conjugated CD4 and PerCP-conjugated CD8 monoclonal antibodies. Splenic T cells were isolated by using BD IMag anti-mouse CD4^+^ and CD8^+^ magnetic beads through positive selection under magnetic field. Isolated splenic T cells stained with PEcy7 conjugated CD25 monoclonal antibody to detect T_reg_ cells. Purity of isolated CD4^+^ and CD8^+^ cells was found to be 85–90%. For T_reg_ inhibition study, we isolated only CD4^+^ T cells using anti-CD4-coated magnetic beads. All the samples were run on BD-FACSCANTO II flowcytometer. Data of ten thousand events were collected and analysed using BD-FACSDiva software.

### Real time PCR

Total RNA was extracted from isolated splenic CD4^+^ T cells with TRIzol reagent followed by cDNA preparation using high capacity cDNA reverse transcription kit. Quantitative real time PCR (RT-PCR) was performed on the QuantStudio 6 Flex instrument with SYBR Green for *FoxP3* gene. *Hypoxanthine-guanine phosphor ribosyl transferase* (*HPRT*) was used for normalization.

### Immunoblot analysis

Protein was isolated from whole tissue lysate of thymus and spleen following standard protocol and estimated using Bradford reagent (Bio-Rad). Protein samples (100 µg) were mixed with loading buffer (5x), ran on 10% SDS-PAGE, transferred to PVDF membrane (Pall Corporation, USA) and probed overnight with Anti-ZFP67 and Anti-RUNX3 rabbit polyclonal antibody (Sigma), FoxP3 mouse monoclonal antibody (Merck-Millipore, USA) at 4 °C and developed with HRP-conjugated goat anti-rabbit and anti-mouse IgG (Santa Cruz Biotech, CA, USA) using chemi-luminescence substrate (Merck-Millipore, USA) under ImageQuant LAS 500 system (GE Healthcare, USA). The densitometric analysis was done using Image-J. Band intensities were normalized with GAPDH band intensity. Primary antibodies were used in 1:2000 and secondary antibodies were used in 1:3000 dilution.

### Cytokine assay

Splenocytes were seeded at a density of 0.1 × 10^6^ cells/200 µl/ well in 96 well culture plates and incubated in the absence or presence of concanavalin A (ConA) (2.5 µg/ml; Sigma) in a 5% CO_2_ incubator at 37 °C for 72 h. Culture supernatants were collected and pooled from each treatment group, and secreted cytokines were measured in supernatant plated in triplicate using a Milliplex mouse cytokine assay kit as previously described^59^ in Bio-Plex MAGPIX multiplex reader (Bio-Rad). Fluorescence associated with different antibody-coated magnetic beads was read to quantitate respective cytokines. The T_h_1 and T_h_2 related cytokines measured were Interferon-γ (IFNγ), IL12, Tumor necrosis factor-α (TNFα), IL4, IL5, IL10 and Tumor growth factor- β (TGF β).

### M. fortuitum infection for resistance assay

Following 30 day of exposure to 0.38 ppm arsenic, mice were injected via tail vein with 10^7^ bacteria per 100 µl of PBS. After 1,3,7,15 and 21 days post infection (DPI) (Silva *et al*. 2010) kidney was aseptically removed and homogenized in normal saline with 0.1% tween 20. Bacterial load in kidney was determined by counting colony forming unit (CFU) on OADC (oleic acid, albumin, dextrose, catalase; Difco) supplemented Middlebrook 7H10 agar (Difco) plates. Colonies were counted after 3 days of incubation at 37 °C. All the microbiological works were done at CSIR-Central Drug Research Institute, India.

### Histopathology

Thymus and spleen from control and 30 day arsenic treated mice were isolated from heparinized-PBS perfused mice and fixed in 4% paraformaldehyde for 48 h at 4 °C. Paraformaldehyde-fixed tissues were dehydrated through graded alcohol, embedded in paraffin and routine microtomy carried out to obtain 5 µm thick sections. The sections were stained with hematoxylin and eosin followed by microscopic examination.

### Biochemical analysis of serum

Blood samples collected through cardiac puncture were allowed to clot at room temperature for about 60 minutes. Samples were centrifuged at 2000 g for 15 minutes at 4 °C and serum collected in micro-centrifuge tube. Levels of aspartate aminotransferase (AST), alanine aminotransferase (ALT) and urea in serum were measured in an Automated Biochemical Clinical Auto Analyzer (ChemWell, USA) using kit from SPINREACT (Spain).

### Statistical analysis

Statistical analysis was done by GraphPad Prism (GraphPad Software Inc., San Diego, CA) using One way analysis of variance (ANOVA) followed by Newman-Keuls test with 95% confidence intervals and p value < 0.05 was considered significant.

## Electronic supplementary material

Supplementary information
