# Supplementary material for: Arsenic exposure impels CD4 commitment in thymus and suppress T cell cytokine secretion by increasing regulatory T cells
Source: Sci Rep. 2017 Aug 2;7:7140. doi: 10.1038/s41598-017-07271-z (PMC5541098; doi:10.1038/s41598-017-07271-z)
Supplement: Supplementary file 1 — Supplementary information [file 41598_2017_7271_MOESM1_ESM.pdf]

**Supplementary Information**

**Title**

Arsenic exposure impels CD4 commitment in thymus and suppresses T cell cytokine secretion by increasing regulatory T cells

**Name of authors**

Ruchi Gera[1,2],Vikas Singh[1,2], Sumonto Mitra[1], Anuj Kumar Sharma[1], Alok Singh[3], Arunava Dasgupta[3], Dhirendra Singh[4], Mahadeo Kumar[4], Pankaj Jagdale[4], Satyakam Patnaik[5] and Debabrata Ghosh[*1]

**The affiliation(s) and address (es) of the author(s)**

[1]Immunotoxicology Laboratory, Food, Drug & Chemical Toxicology Group, CSIR-Indian Institute of Toxicology Research (CSIR-IITR), Vishvigyan Bhavan, 31 Mahatma Gandhi Marg, Lucknow-226001, Uttar Pradesh, India

[2]Academy of Scientific and Innovative Research (AcSIR), CSIR-IITR campus, Lucknow-226001, India

[3]Microbiology, CSIR- Central Drug Research Institute, Lucknow, Uttar Pradesh, India

[4] Regulatory toxicology, CSIR-Indian Institute of Toxicology Research, Lucknow, Uttar Pradesh, India

[5]Water Analysis Laboratory, Nanotherapeutics and Nanomaterial Toxicology Group, CSIR-Indian Institute of Toxicology Research, Lucknow, Uttar Pradesh, India

***To whom correspondence should be addressed**: debabrata.ghosh@iitr.res.in; debabrataghosh78@gmail.com; Debabrata Ghosh, Immunotoxicology Laboratory, Food Drug and Chemical toxicology group and Nanotherapeutics & Nanomaterial toxicology Group, CSIR-Indian Institute of Toxicology Research (CSIR-IITR), Vishvigyan Bhawan, 31, M. G. Marg, P.O. Box No. 80, Lucknow-226001, Uttar Pradesh, India

**Tel**:+915222627586;**Ext**.252;**Fax**:+91522228227,

**Arsenic exposure promoted influx of DN cells into thymus from bone marrow:**

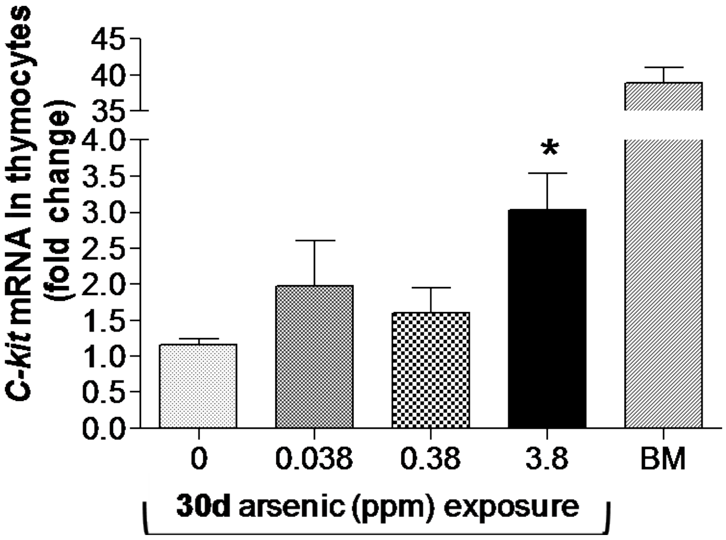


**Supplementary Figure S1. Arsenic-induced alteration in *c-kit* expression in thymocyte**. Total RNA was extracted from thymus tissue of all 30 days arsenic-exposed groups. *c-kit* mRNA level was measured under QuantStudio 6 Flex instrument with SYBR Green by normalizing with *Hprt*. Bone marrow (BM) cells were isolated from femur bone of mice and used as a positive control for c-kit expression. Values are mean±S.E. (n=3 per group). *$p<0.05$ compared to control using one way ANOVA followed by Newman-Keuls test with 95% confidence intervals.

## Arsenic promoted percentage of $CD4^+$ cells without inducing apoptosis in $CD8^+$ cells:

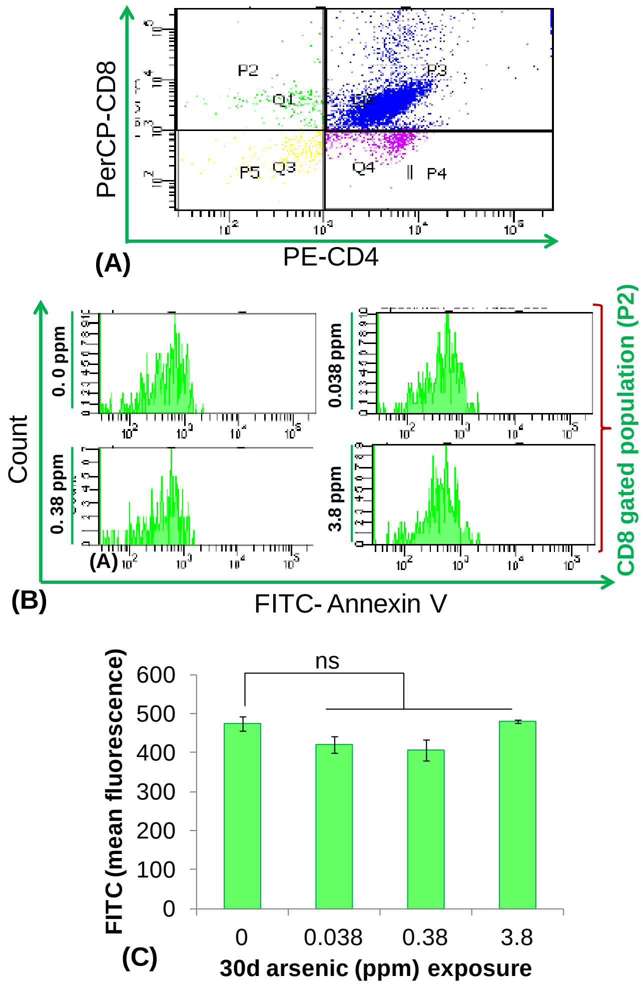


**Supplementary Figure S2. Arsenic-induced alteration in Annexin-FITC labeling in thymic $CD8^+$ cells**. Thymocyte suspension was prepared from thymus obtained from 30 days arsenic-exposed mice. Thymocytes ($0.2x10^6$ cells) were stained with PE and PerCP conjugated anti-CD4 and anti-CD8 antibodies respectively along with FITC conjugated annexin-V for 1hr, run in BD-FACSCANTO II and analyzed using FACS-Diva software. **(A)** Representative dot plot showing the strategy used to gate $CD8^+$ cells as P2 population (green color) in thymocyte suspension. **(B)** Representative histograms showing annexin-V staining of CD8 gated cells. **(C)** Bar diagram reflecting the mean fluorescence of FITC conjugated with annexin-V in $CD8^+$ cells. Values are mean±S.E. (n=3 per group). ns = not significant when compared to control using One way ANOVA followed by Newman-Keuls test with 95% confidence intervals.

**Original full-length western blots of Thpok, RunX3 and FoxP3 with corresponding GAPDH (loading control):**

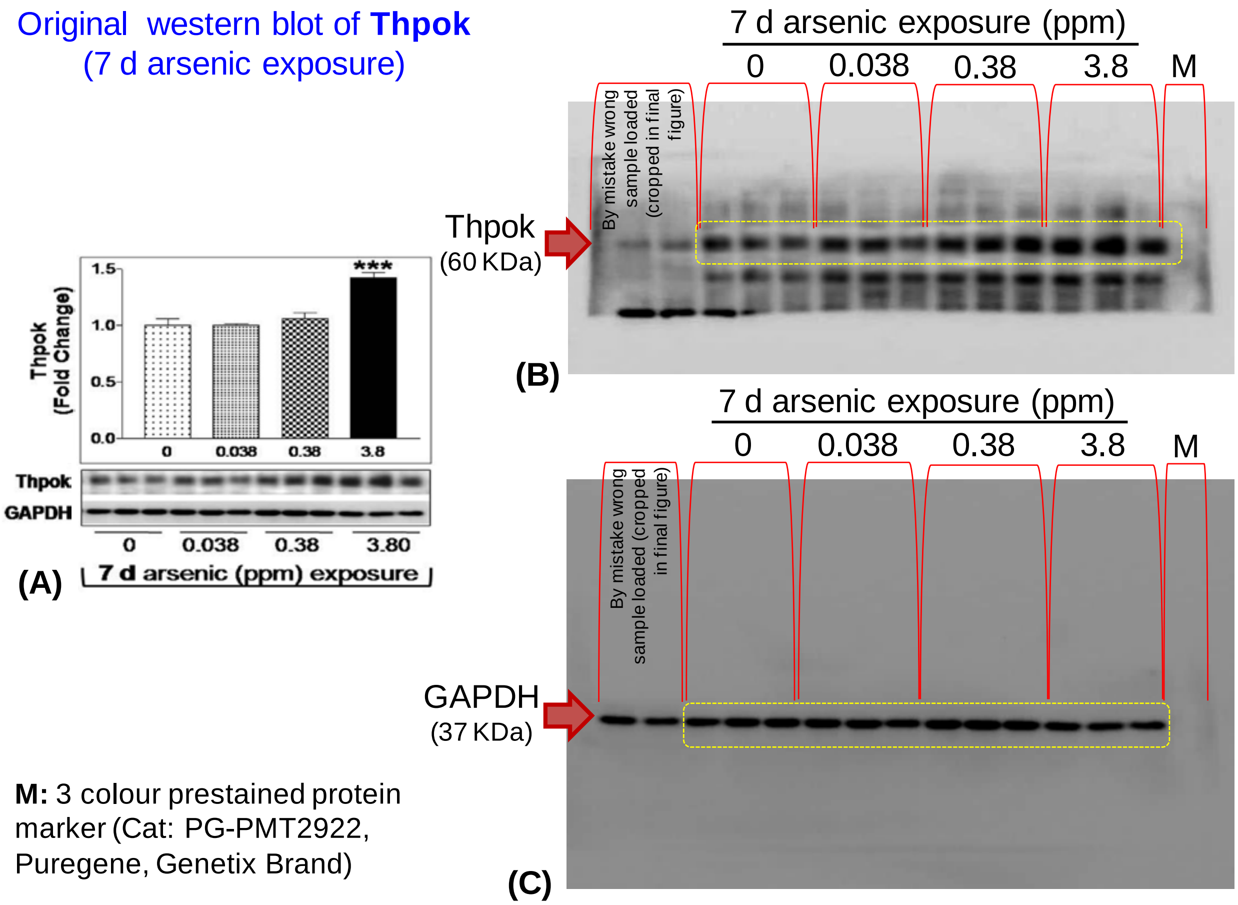


**Supplementary Figure S3: Arsenic-induced alteration in Thpok expression following 7 days arsenic exposure.** Thymus dissected from mice treated with arsenic (0.00, 0.038, 0.38 and 3.8 ppm) orally for 7days were homogenized for protein preparation and examined for the expression of **(A)** Thpok and GAPDH. Full-length blot for **(B)** Thpok and, **(C)** corresponding GAPDH.

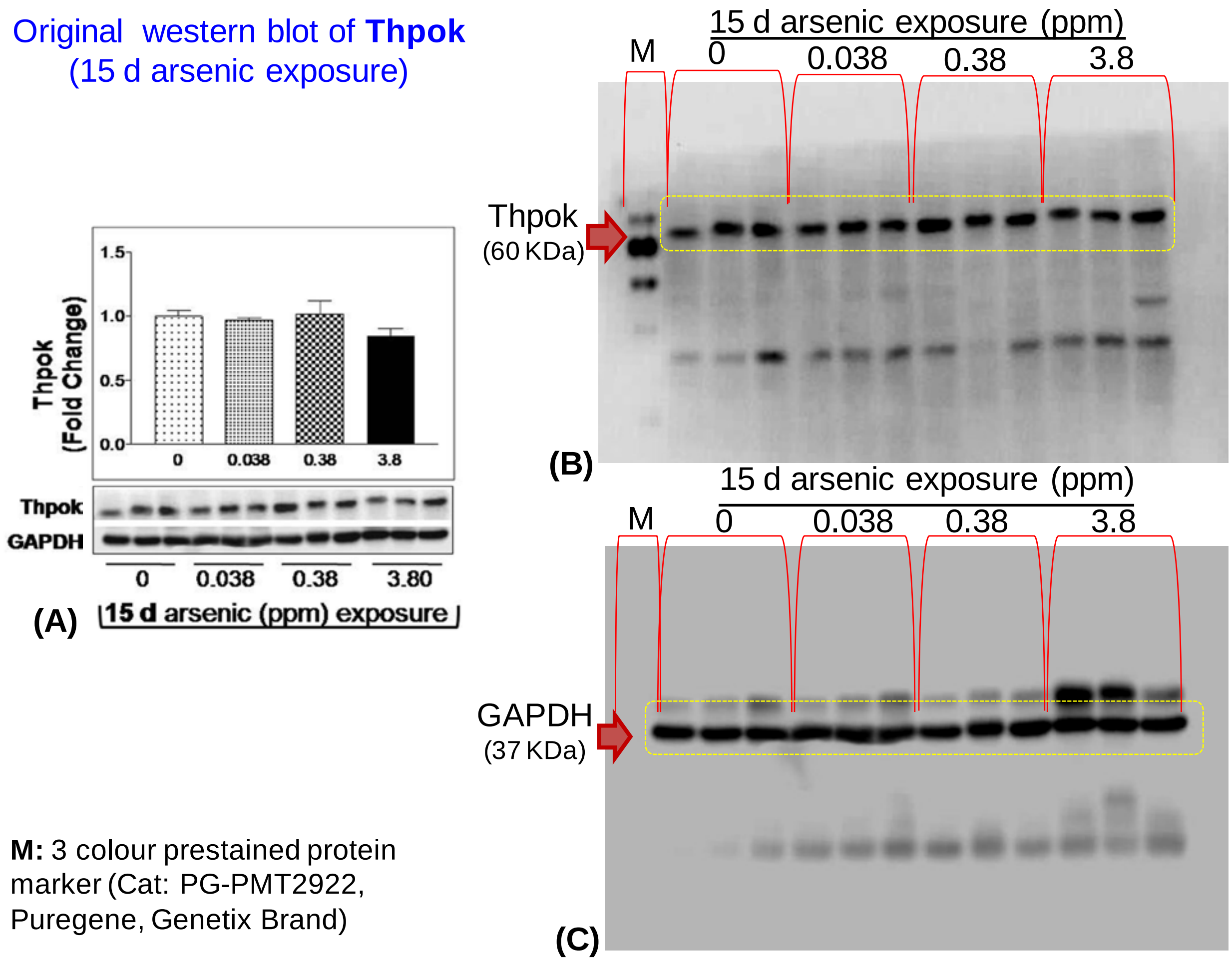


**Supplementary Figure S4: Arsenic-induced alteration in Thpok expression following 15 days arsenic exposure.** Thymus dissected from mice treated with arsenic (0.00, 0.038, 0.38 and 3.8 ppm) orally for 15 days were homogenized for protein preparation and examined for the expression of **(A)** Thpok and GAPDH. Full-length blot for **(B)** Thpok and, **(C)** corresponding GAPDH.

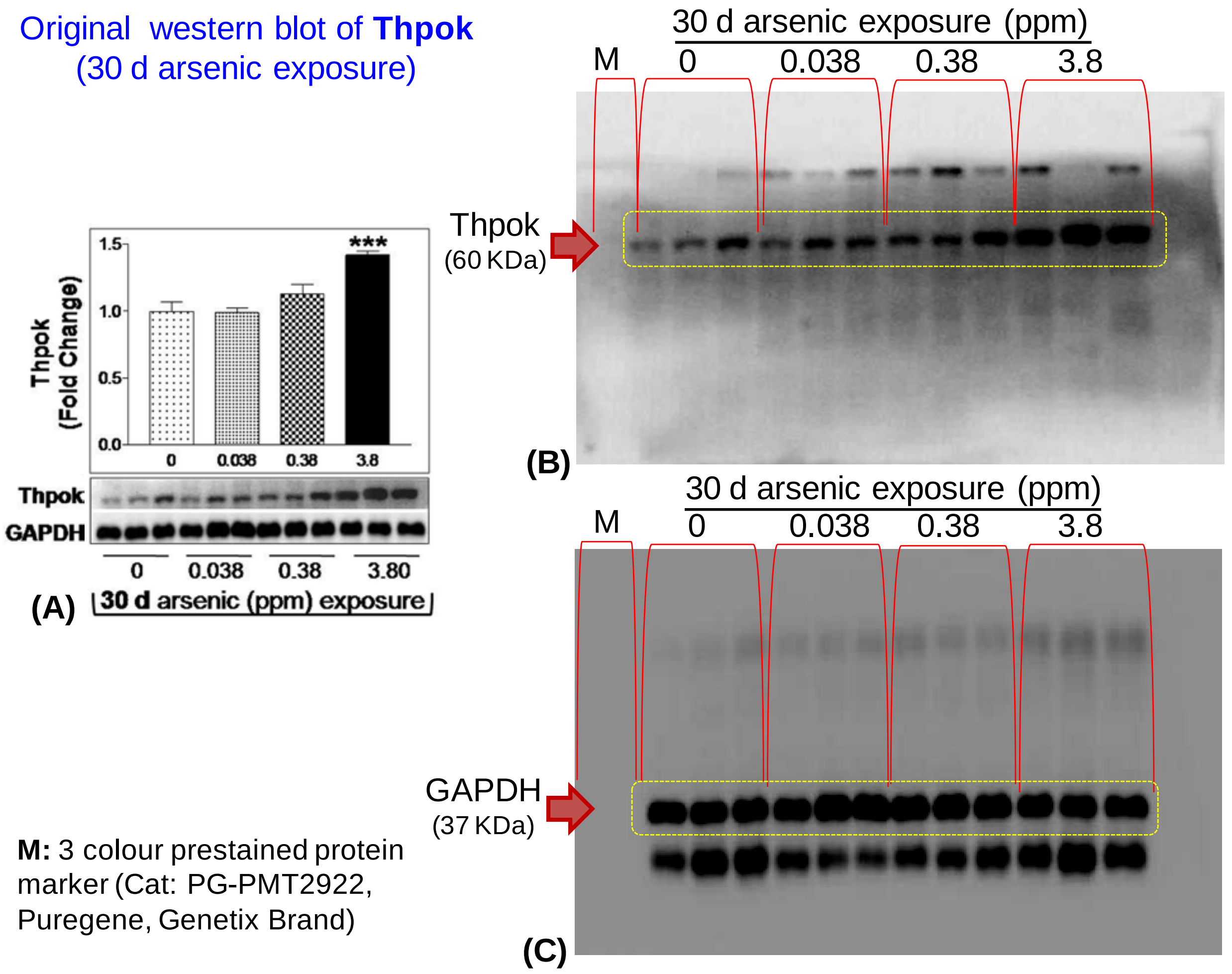


**Supplementary Figure S5: Arsenic-induced alteration in Thpok expression following 30 days arsenic exposure.** Thymus dissected from mice treated with arsenic (0.00, 0.038, 0.38 and 3.8 ppm) orally for 30 days were homogenized for protein preparation and examined for the expression of **(A)** Thpok and GAPDH. Full-length blot for **(B)** Thpok and, **(C)** corresponding GAPDH.

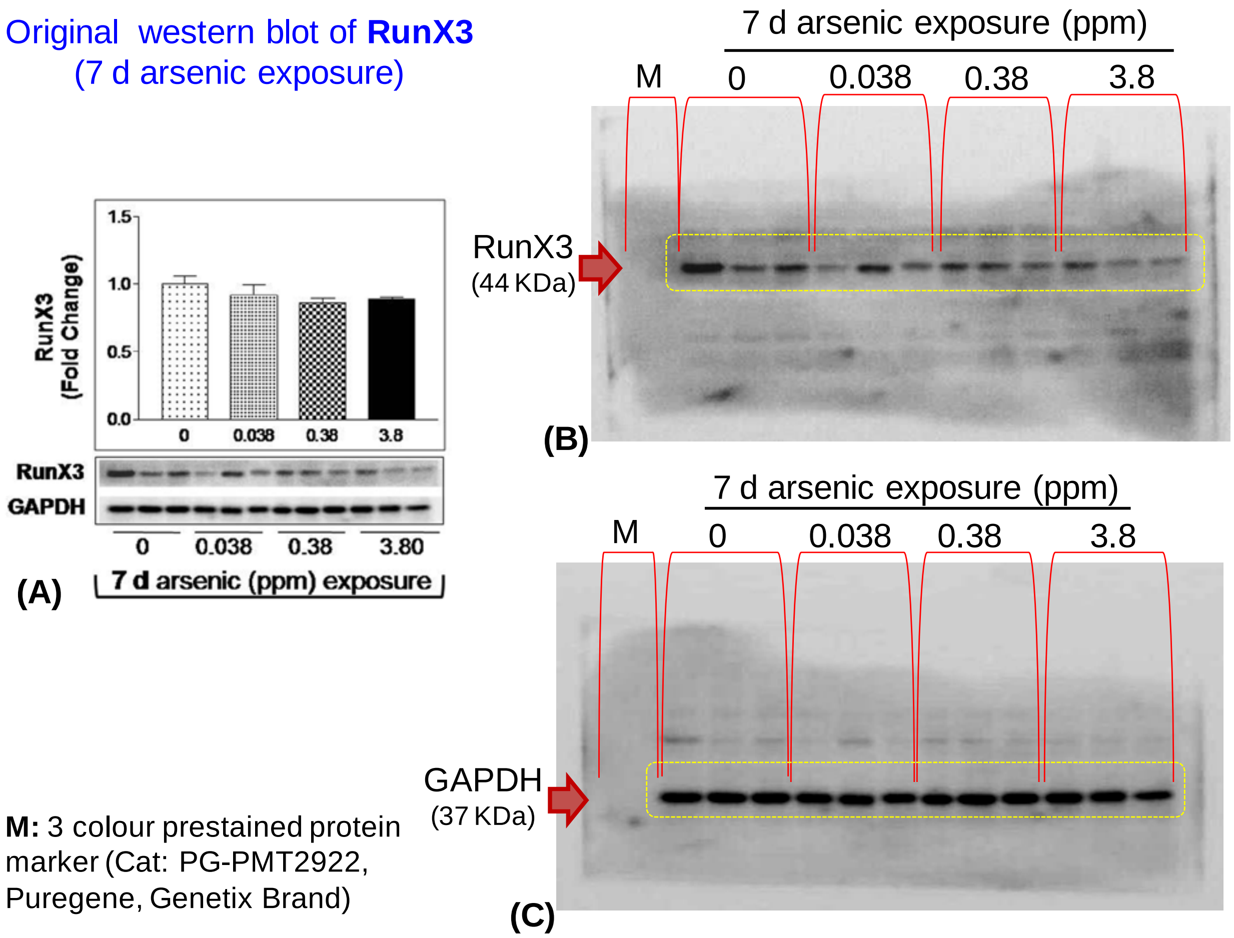


**Supplementary Figure S6: Arsenic-induced alteration in RunX3 expression following 7 days arsenic exposure.** Thymus dissected from mice treated with arsenic (0.00, 0.038, 0.38 and 3.8 ppm) orally for 7 days were homogenized for protein preparation and examined for the expression of **(A)** RunX3 and GAPDH. Full-length blot for **(B)** RunX3 and, **(C)** corresponding GAPDH.

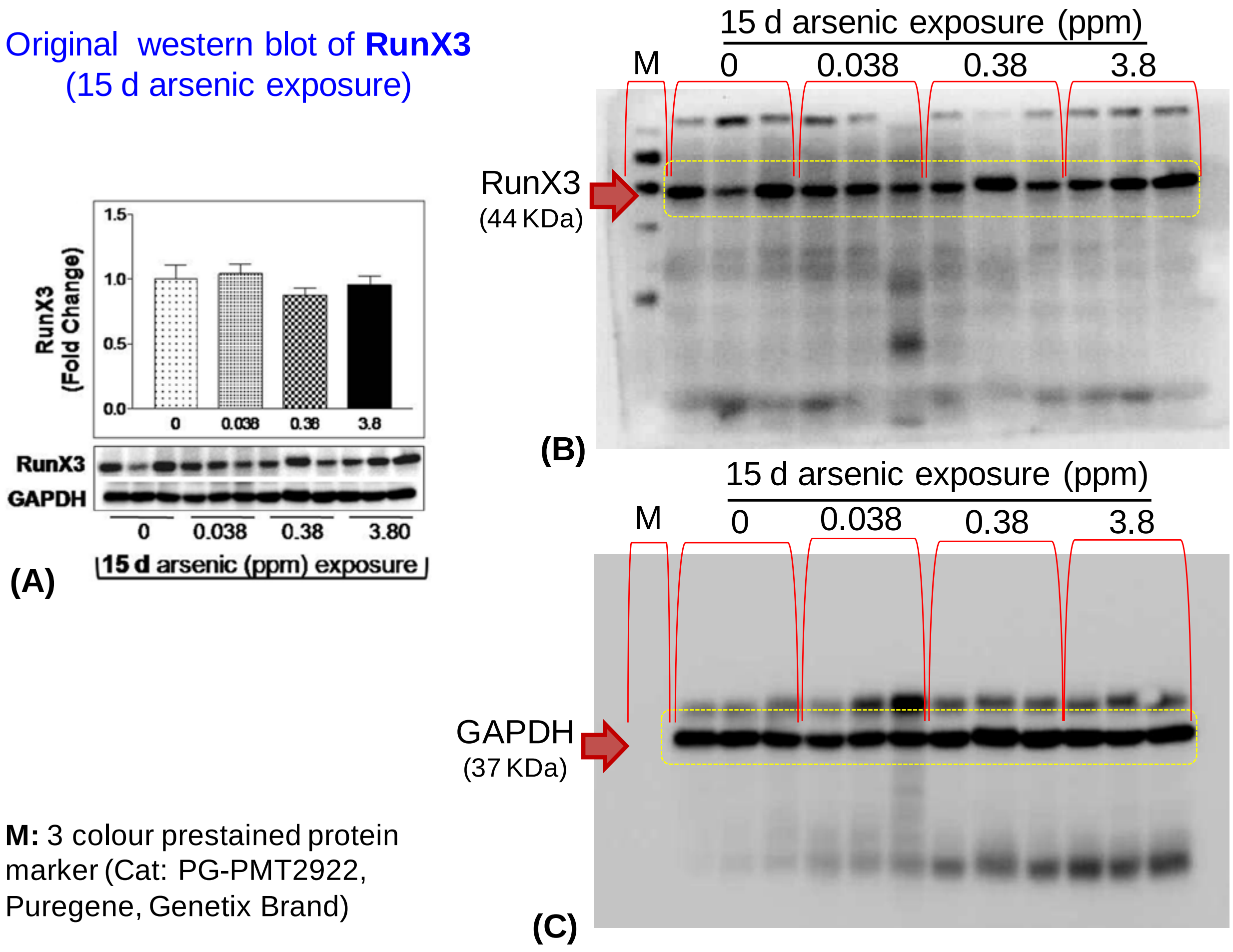


**Supplementary Figure S7: Arsenic-induced alteration in RunX3 expression following 15 days arsenic exposure.** Thymus dissected from mice treated with arsenic (0.00, 0.038, 0.38 and 3.8 ppm) orally for 15 days were homogenized for protein preparation and examined for the expression of **(A)** RunX3 and GAPDH. Full-length blot for **(B)** RunX3 and, **(C)** corresponding GAPDH.

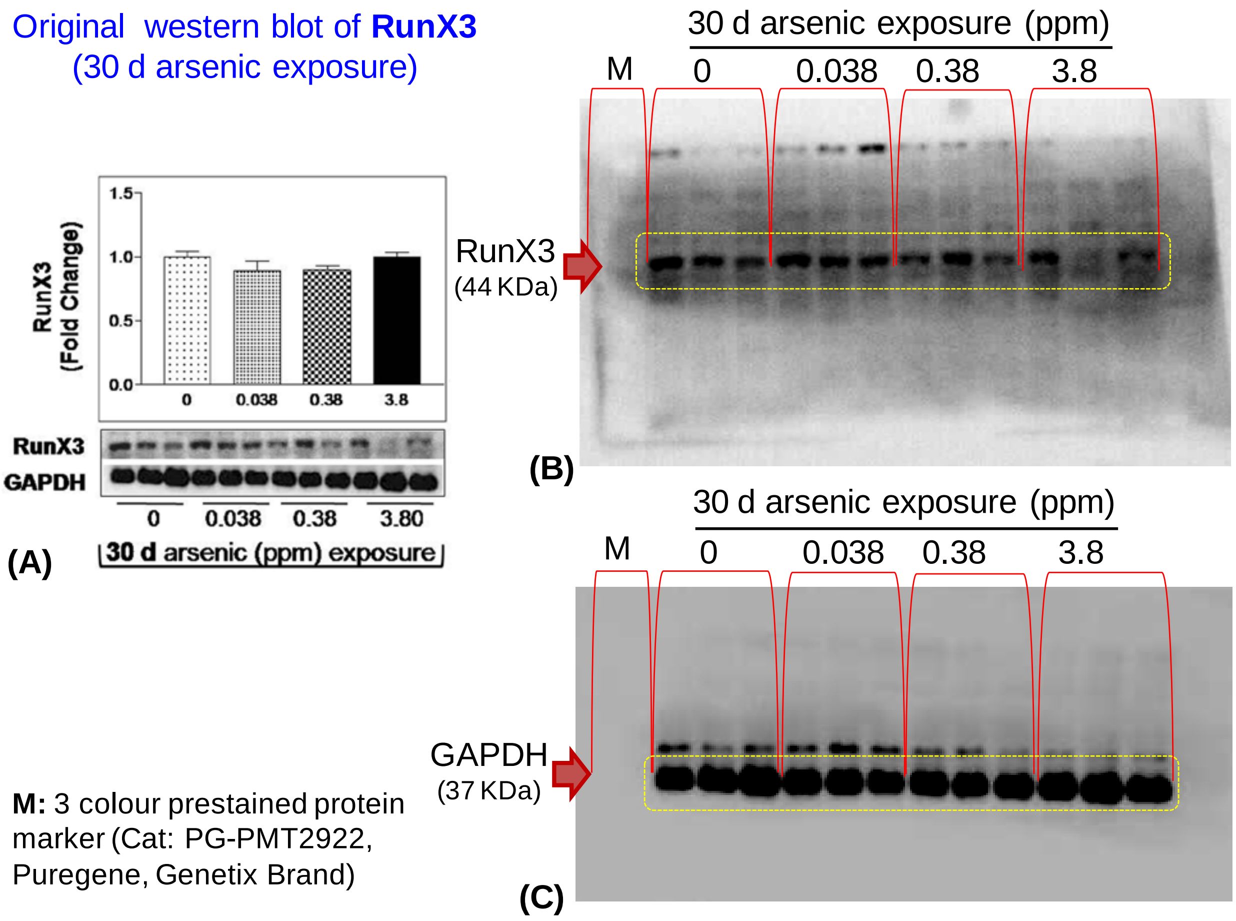


**Supplementary Figure S8: Arsenic-induced alteration in RunX3 expression following 30 days arsenic exposure.** Thymus dissected from mice treated with arsenic (0.00, 0.038, 0.38 and 3.8 ppm) orally for 30 days were homogenized for protein preparation and examined for the expression of **(A)** RunX3 and GAPDH. Full-length blot for **(B)** RunX3 and, **(C)** corresponding GAPDH.

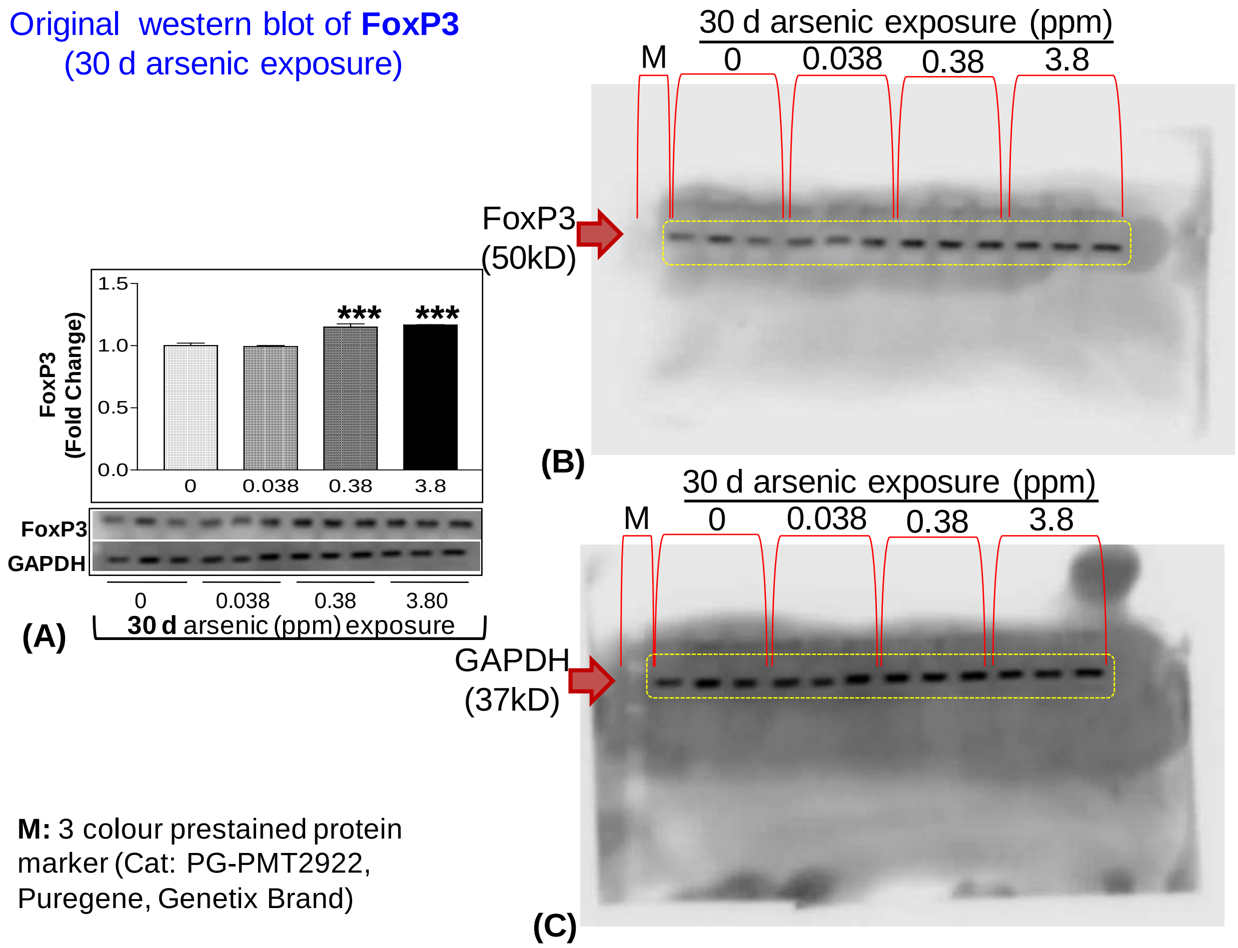


**Supplementary Figure S7: Arsenic-induced alteration in FoxP3 expression following 30 days arsenic exposure.** Spleen dissected from mice treated with arsenic (0.00, 0.038, 0.38 and 3.8 ppm) orally for 30 days were homogenized for protein preparation and examined for the expression of **(A)** FoxP3 and GAPDH. Full-length blot for **(B)** FoxP3 and, **(C)** corresponding GAPDH.
